# Design parallel and sequential quantum algorithms via spectral element method on distributed quantum architectures

**DOI:** 10.1038/s41598-025-28546-w

**Published:** 2025-12-29

**Authors:** Amir Hossein Salehi Shayegan

**Affiliations:** Faculty of Mathematics, K. N. Toosi University of Technology, Tehran, Iran

**Keywords:** Distributed quantum computing, Spectral element method, Quantum algorithms, Mathematics and computing, Physics

## Abstract

In this work, we present a solution to the critical limitation of qubit capacity in near-term quantum hardware by giving a hybrid framework that integrates the spectral element method (SEM) with distributed quantum computing. Using domain decomposition techniques, the additive and multiplicative Schwarz methods, the global problem is divided into smaller subproblems that each require significantly fewer qubits, making them suitable for current quantum devices. The HHL algorithm is applied locally to these subproblems, enabling quantum speedups without exceeding hardware constraints. Numerical results validate the approach, demonstrating a scalable and practical solution for high-accuracy simulations despite qubit limitations.

## Introduction

Consider the parabolic problem:

1 $$\begin{aligned} {u_t} - u_{xx}= f(x,t),\,\,\,\,\,\,\,\,\,\,\,\,\,\,\,\,\,\,\,\,\,\,\,\,\,\,\,\,\, \,\,\,\,\,\,\,\,\,\,\,\,\,\,\,\,\,\,\, (x,t) \in {Q_T}: = \Omega \times (0,T),\end{aligned}$$

2 $$\begin{aligned} u(0,t) =u(1,t)= 0,\,\,\,\,\,\,\,\,\,\,\,\,\,\,\,\,\,\,\,\,\,\,\,\,\,\,\,\,\,\,\,\,\,\,\,\,\,\,\,\,\,\,\,\,\, t \in (0,T), \,\,\,\,\,\,\,\,\, \,\,\,\,\,\,\,\,\,\,\,\,\,\,\, \,\,\,\,\,\,\,\,\,\,\,\,\,\,\, \end{aligned}$$

3 $$\begin{aligned} u(x,0) = \phi (x),\,\,\,\,\,\,\,\,\,\,\,\,\,\,\,\,\,\,\,\,\,\,\,\,\,\,\,\,\,\,\,\,\,\,\,\,\,\,\,\,\,\,\,\,\,\,\,\,\,\,\,\,\,\,\,\,\,\,x \in \Omega ,\,\,\,\,\,\,\,\,\,\,\,\,\,\,\,\,\,\,\,\,\,\,\,\,\,\,\,\,\,\,\,\,\,\,\,\,\,\,\,\,\,\,\,\,\,\,\,\,\,\, \end{aligned}$$

where $\Omega :=(0,1)$, $f=f(x,t)\in {L_2}(Q_{T})$ and $\phi =\phi (x) \in {L_2}(\Omega )$. The numerical solution of partial differential equations plays a crucial role in modeling and simulating complex physical systems in fields such as fluid dynamics, electromagnetics, and structural mechanics. Among high-accuracy discretization techniques, the spectral element method has gained prominence for its ability to achieve exponential convergence rates while maintaining geometric flexibility^1–4^. SEM combines the advantages of spectral methods and finite element methods by partitioning the domain into elements and employing high-degree polynomial basis functions within each element. Despite its accuracy, the global linear systems arising from SEM discretizations can be computationally intensive, especially for high-resolution or multidomain problems.

Meanwhile, quantum computing offers the potential to transform numerical linear algebra by providing speedups for certain classes of problems. In particular, the Harrow-Hassidim-Lloyd (HHL) algorithm enables the quantum solution of linear systems of equations with exponential complexity improvements under specific conditions^5^. These features make HHL a compelling candidate for accelerating the solution of the linear systems that emerge from SEM discretizations. However, the practical realization of quantum algorithms such as HHL is currently constrained by the hardware limitations of near-term quantum devices, often referred to as Noisy Intermediate-Scale Quantum (NISQ) systems. These devices typically support only a limited number of coherently controllable qubits, which restricts the size and complexity of problems that can be processed quantum-mechanically in a single execution. Moreover, quantum gates are inherently error-prone, and the accumulation of gate errors and decoherence effects severely limits the circuit depth and algorithmic fidelity achievable on current machines. As a result, executing large-scale or high-precision computations solely on quantum hardware is presently infeasible. These limitations have prompted the development of hybrid quantum-classical approaches, where the quantum processor is used for specific subroutines, such as solving linear systems or performing eigendecompositions, while the classical processor manages overall data orchestration, pre/post-processing, and the parts of the algorithm that do not benefit from quantum speedup. This division of labor is essential for realizing practical quantum advantage in computational science, especially when integrating quantum solvers with large-scale classical methods such as the spectral element method.

To overcome these challenges, this work proposes an integrated framework that couples distributed quantum computing with SEM. Distributed quantum computing leverages multiple interconnected quantum processors (nodes) to collaboratively perform quantum computations beyond the capacity of a single device^6,7^. By distributing the workload across quantum nodes and using entangled communication channels, the framework facilitates scalable quantum computation. Within this architecture, we design parallel and sequential quantum algorithms that match the decomposition structure of SEM, enabling efficient hybrid execution.

A core innovation in our approach is the use of domain decomposition methods, namely the additive Schwarz method and the multiplicative Schwarz method, tailored for quantum implementation^8^. The additive Schwarz method lends itself to parallel execution, as each subdomain problem can be solved independently, which aligns well with parallel quantum subroutines running on separate quantum nodes. On the other hand, multiplicative Schwarz method uses a sequential update mechanism, making it well-suited for sequential quantum execution with state reuse and entanglement-aware scheduling. These methods help improve convergence when using quantum linear solvers such as HHL and balance computational cost with inter-node communication.

By integrating quantum speedups through the HHL algorithm with the spectral accuracy of SEM and the parallelism of additive Schwarz method and multiplicative Schwarz method, the proposed framework aims to deliver scalable and high-performance solvers for complex physical simulations. Preliminary numerical experiments indicate that the framework not only improves computational efficiency but also adapts well to near-term quantum architectures. This work contributes to bridging classical high-order numerical methods with emerging quantum technologies, paving the way for future advances in scientific computing.

This paper is structured as follows. Section "Spectral element method" provides an overview of the spectral element method and its mathematical foundations. Section "Distributed quantum computing framework" introduces the distributed quantum computing framework and details the implementation of the additive and multiplicative Schwarz methods as domain decomposition strategies. In Sect. "Parallel and sequential quantum algorithm design", we present the proposed parallel and sequential quantum algorithms developed within this hybrid framework. Finally, Sect. “Numerical results” illustrates the effectiveness of the proposed approach through a numerical example, demonstrating its computational efficiency and scalability.

## Spectral element method

In this section, we discuss the spectral element method, a high-order numerical technique for solving partial differential equations. SEM combines the strengths of two established methods: the finite element method (FEM) and the spectral method (SM). The high accuracy of SEM is inherited from the spectral approach, while its flexibility and geometric adaptability stem from the finite element framework^1,9,10^. In the following, we provide a brief overview of this method.

The spectral element method begins with the spatial discretization of the problem. To achieve this, we first multiply both sides of equation (1) by a test function $v \in H_0^1(\Omega )$, and then integrate over the domain $\Omega$ to obtain:

4 $$\begin{aligned} \int _\Omega {{u_t}(x,t)v(x)dx} + \int _\Omega {\nabla u(x,t) \cdot \nabla v(x)dx} = \int _\Omega {f(x,t) v(x)dx}. \end{aligned}$$

The second step of SEM involves partitioning the model domain $\Omega$ into subdomains. Similar to FEM, the domain is divided into $n_e$ non-overlapping subdomains (elements) denoted by $\Omega _e$:

$$\begin{aligned} \bar{\Omega }= \bigcup \limits _{e = 1}^{{n_e}} {{{\bar{\Omega }}_e}},\,\,\,\,\,\,\,\,\,\bigcap \limits _{e = 1}^{{n_e}} {{\Omega _e} = \emptyset .} \end{aligned}$$

Hence, the integrals in equation (4) can be evaluated independently over each subdomain, so one can write:

5 $$\begin{aligned} \int _{\Omega _{e}} {{u_t}(x,t)v(x)dx} + \int _{\Omega _{e}} {\nabla u(x,t) \cdot \nabla v(x)dx} = \int _{\Omega _{e}} {f(x,t)v (x)dx}, \end{aligned}$$

for $e=1,2,\ldots ,n_{e}$. Then, each integral in equation (5) is approximated using the Gauss-Lobatto-Legendre (GLL) quadrature. To do this, each element is mapped onto the standard reference interval $[-1, 1]$. In this method, the collocation points are defined as:

6 $$\begin{aligned} \xi _0 = -1,\quad \xi _i = \text {zeroes of } P_N&,\quad \xi _N = 1,\quad 1 \le i \le N-1, \end{aligned}$$

where

$$\begin{aligned} P_N(x) = \frac{1}{2^N N!} \frac{d^N}{dx^N}(x^2 - 1)^N, \end{aligned}$$

is the Legendre polynomial of degree $N$. The corresponding weights for the numerical integration are given by

$$\begin{aligned} w_i = \frac{2}{N(N+1)} \frac{1}{\left( P_N(\xi _i)\right) ^2}, \quad i = 0, 1, \ldots , N. \end{aligned}$$

For more details, see^10^. Now, if in equation (5) we approximate the solution as:

$$\begin{aligned} u(x,t) \simeq \bar{u}(x,t) = \sum \limits _{i = 0}^N c_i^{(e)}(t)\, \ell _i^{(e)}(x), \qquad x \in \Omega _{e}, \end{aligned}$$

and choose the test function as

$$\begin{aligned} v = \ell _j^{(e)}(x), \qquad x \in \Omega _{e}, \quad j = 0, 1, \ldots , N, \end{aligned}$$

then we obtain the following semi-discrete formulation for $j = 0, 1, \ldots , N$:

7 $$\begin{aligned} \sum \limits _{i = 0}^N \dot{c}_i^{(e)}(t) \left( \int _{\Omega _e} \ell _i^{(e)}(x) \, \ell _j^{(e)}(x) \, dx \right) + \sum \limits _{i = 0}^N c_i^{(e)}(t) \left( \int _{\Omega _e} \nabla \ell _i^{(e)}(x) \cdot \nabla \ell _j^{(e)}(x) \, dx \right) = \int _{\Omega _e} f(x,t) \, \ell _j^{(e)}(x) \, dx. \end{aligned}$$

**Fig. 1:**
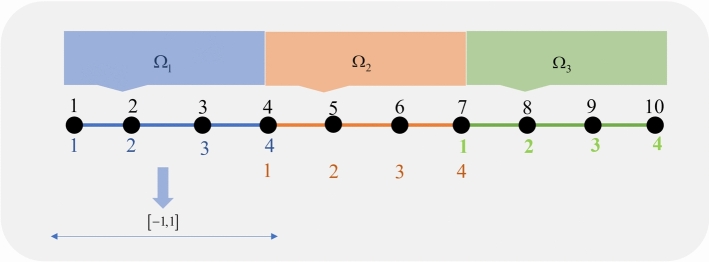
Decomposition and mapping. Here the model is divided into three elements $n_{e}=3$ and also the degree of Legendre polynomial is $N=3$.

We note that $\ell _i^{(e)}(x)$, for $i = 0, 1, \ldots , N$, are the Lagrange interpolating polynomials associated with the $N+1$ nodes:

8 $$\begin{aligned} \chi _i^{(e)} = N^{(e)}(\xi _i), \end{aligned}$$

where $N^{(e)}(\cdot )$ is the mapping function that transforms the reference nodes $\xi _i$, $i = 0, 1, \ldots , N$, on the standard interval $[-1, 1]$ to the corresponding nodes on the physical element $\Omega _e$. Figure 1 illustrates the idea of dividing the model domain into elements on the basis of a one-dimensional string. Each element domain is then transformed as shown for element number 1.

To approximate the first integral in equation (7) using GLL quadrature, we write:

$$\begin{aligned} m_{i,j}^{(e)} := \int _{\Omega _e} \ell _i^{(e)}(x)\,\ell _j^{(e)}(x)\,dx = \int _{-1}^{1} \ell _i^{(e)}(\xi )\,\ell _j^{(e)}(\xi )\,J(\xi )\,d\xi \simeq \sum _{k=0}^{N} w_k\,\ell _i^{(e)}(\xi _k)\,\ell _j^{(e)}(\xi _k)\,J(\xi _k), \end{aligned}$$

where $J(\xi )$ denotes the Jacobian of the transformation. Similarly, for the second and third integrals, we have:

$$\begin{aligned} K_{i,j}^{(e)} := \int _{\Omega _e} \,\nabla \ell _i^{(e)}(x) \cdot \nabla \ell _j^{(e)}(x)\,dx = \int _{-1}^{1} \nabla \ell _i^{(e)}(\xi ) \cdot \nabla \ell _j^{(e)}(\xi )\,J(\xi )\,d\xi \simeq \sum _{k=0}^{N} w_k\,\nabla \ell _i^{(e)}(\xi _k) \cdot \nabla \ell _j^{(e)}(\xi _k)\,J(\xi _k), \end{aligned}$$

and

$$\begin{aligned} F_j^{(e)}(t) := \int _{\Omega _e} f(x,t)\,\ell _j^{(e)}(x)\,dx = \int _{-1}^{1} f(\xi ,t)\,\ell _j^{(e)}(\xi )\,J(\xi )\,d\xi \simeq \sum _{k=0}^{N} w_k\,f(\xi _k,t)\,\ell _j^{(e)}(\xi _k)\,J(\xi _k). \end{aligned}$$

After computing the matrix form of all integrals within each element individually, the next step is the **assembly process**. In the case of one-dimensional elements, we begin by defining a *local-to-global node mapping* (denoted as LtoG), which assigns the local node indices of each element to the corresponding global node indices in the mesh. For example, as illustrated in Fig. 1, the local-to-global node mapping matrix is given by:
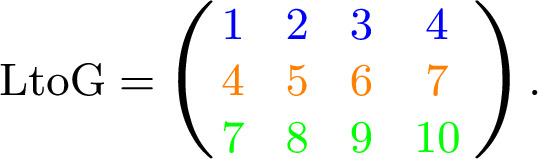


Each row corresponds to the local node numbering of an element and each entry represents the associated global node number. There are $n_{e}$ rows and $N+1$ columns in the LtoG matrix. The $i$th row contains the global node numbers associated with the $i$th element. Using this mapping, the global matrices *M*, *K* and *F* are assembled by looping over each element and adding contributions of the local matrices to the corresponding global entries. Shared nodes naturally accumulate contributions from multiple elements. This procedure ensures that the assembled global matrices correctly represent the entire domain and can then be used in the solution of the finite element system (Algorithm 1).

**Algorithm 1:**
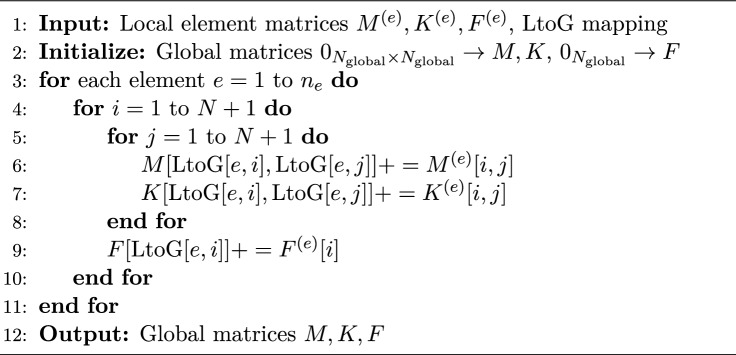
Global Matrix Assembly using LtoG Mapping

Using the assembly rule^11^ and the LtoG matrix, we ultimately obtain the following system of equations (Algorithm 1):

9 $$\begin{aligned} M\dot{C}(t) + KC(t) = F(t), \end{aligned}$$

where, for $n_{e} = 3$ and $N = 3$ (see Fig. 1), the system matrices are assembled as follows:
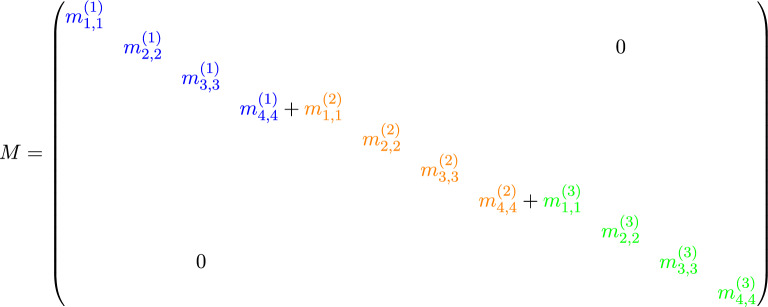

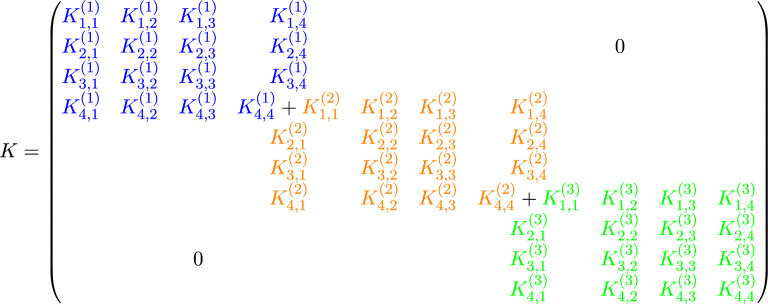

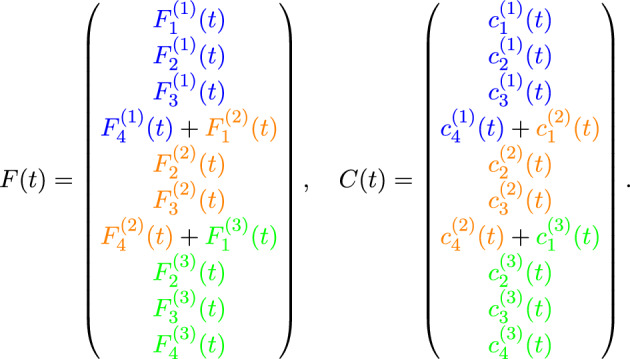


We solve the system of ordinary differential equations (9) using the backward Euler method (Algorithm 2). We discretize the time interval $[0, T]$ into $N_{t}$ uniform steps of size $\Delta t = \frac{T}{N_{t}}$, and define the discrete time levels by

$$t_n = n\Delta t, \quad \text {for } n = 0, 1, \ldots , N_{t}.$$

Accordingly, we denote the approximate solution and forcing term at time $t_n$ as

$$C^n \approx C(t_n), \quad F^n \approx F(t_n).$$

**Algorithm 2:**
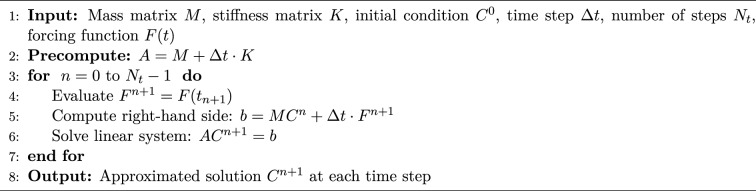
Implicit Backward Euler Method for Solving $M\dot{C}(t) + KC(t) = F(t)$

### Remark 2.1

The backward Euler method is a first-order accurate time integration scheme. It is consistent with local truncation error of order $\mathcal {O}(\Delta t^2)$ and globally convergent with an error of order $\mathcal {O}(\Delta t)$. Due to its unconditional stability (A-stability), the method is particularly effective for stiff systems and guarantees convergence as $\Delta t \rightarrow 0$^12^.

We note that the matrix *A* in Algorithm 2 exhibits the following sparsity pattern and remains constant throughout all iterations:
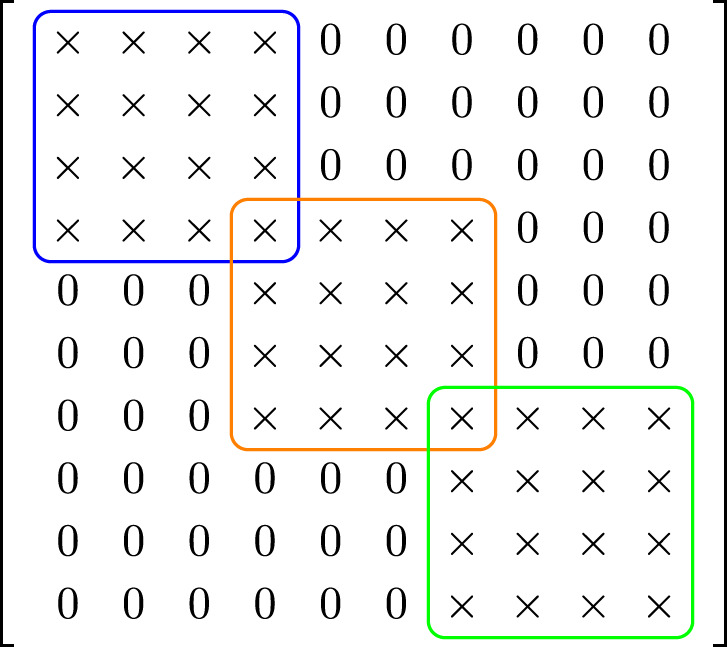


where each “$\times$” denotes a potentially nonzero entry. This block structure exhibits banded and overlapping blocks, which naturally lend themselves to decomposition and parallel processing. Accordingly, we implement this matrix within a **distributed quantum computing framework**, where each subsystem handles a portion of $A$, exploiting its sparsity to optimize quantum resources and improve computational efficiency. This approach effectively addresses the limitation on the number of qubits available in current quantum computers by distributing the problem across smaller quantum subsystems.

## Distributed quantum computing framework

Distributed quantum computing is an emerging paradigm that connects multiple smaller quantum processors (or quantum nodes) through quantum communication channels to collaboratively solve complex computational problems^13,14^. Instead of relying on a single large-scale quantum computer, which remains challenging due to technological and physical constraints, this framework enables scalability by partitioning tasks across interconnected quantum systems^15^. In this approach, quantum information and entanglement are shared between nodes, allowing them to perform joint quantum algorithms and computations that exceed the capability of any individual processor^16^. Distributed quantum computing leverages the combined power of multiple quantum devices, overcoming limitations such as restricted qubit counts, error rates, and coherence times^17^. By decomposing large problems into smaller subproblems handled by separate quantum nodes, distributed quantum computing enhances flexibility, fault tolerance, and resource efficiency. This makes it a promising route toward practical quantum advantage and enables applications in quantum simulation, optimization, and secure communication^18,19^.

### Domain decomposition methods for solving linear system of equations $Ax=b$

When the system size is large, direct solution methods become computationally expensive or infeasible. Domain decomposition methods offer an effective approach to solving such systems by breaking the problem into smaller subproblems associated with subdomains of the original computational domain. The domain decomposition approach consists of the following steps:

1. **Partitioning:** The computational domain is partitioned into for example $m$ overlapping or non-overlapping subdomains. Correspondingly, the global system matrix $A$ and vectors $x$, $b$ are decomposed into local parts associated with these subdomains.
2. **Local subproblems:** For each subdomain, define a restriction operator $R^{(e)}$ that extracts local degrees of freedom from the global system. Then, the local system matrix on subdomain $e$ is
  $$A^{(e)} = R^{(e)} A R^{(e)T},$$
  and the local right-hand side is
  $$b^{(e)} = R^{(e)} b.$$
3. **Iterative Solution:** The global solution $x$ is approximated by iteratively solving these smaller local systems and combining their solutions. Corrections from local solves are propagated across subdomains through overlapping regions or interface conditions.

Two main classes are commonly used, overlapping domain decomposition and non-overlapping domain decomposition. In overlapping methods, subdomains share common regions. This overlap facilitates information exchange and accelerates convergence. Schwarz methods (both additive and multiplicative) are typical examples, where local solves on overlapping subdomains iteratively update the global solution. Also, in non-overlapping methods, subdomains meet only at interfaces without overlap. Interface conditions and constraints are imposed to ensure continuity and compatibility. Examples include the Schur complement methods and the finite element tearing and interconnecting (FETI) methods.

Domain decomposition methods offer several advantages, including inherent parallelization since local subproblems can be solved concurrently, which makes these methods well-suited for distributed computing architectures. They also reduce memory usage and computational cost by breaking a large system into many smaller, more manageable systems that are typically more efficient to solve. Additionally, domain decomposition provides flexibility, allowing different numerical methods or discretizations to be applied to different subdomains according to local problem characteristics. Finally, these methods enhance scalability, enabling the solution of very large problems that would otherwise be computationally intractable.

### Schwarz methods

Schwarz methods^20^ are classical iterative techniques where the solution is updated through sequential or parallel subdomain solves, with information exchanged via overlapping regions. Two principal variants of Schwarz methods exist: the *Additive Schwarz method*, where subdomain corrections are computed simultaneously in parallel and then combined to update the global solution, and the *Multiplicative Schwarz method*, in which subdomain solves are performed sequentially, with each subdomain using the most recently updated global solution before moving to the next. To be exact, we aim to solve the linear system:

$$\begin{aligned} A x = b, \end{aligned}$$

where $A = M + \Delta t \cdot K$. For simplicity, we assume that $A \in \mathbb {R}^{10 \times 10}$ (Fig. 1). Decomposing the matrix as

$$\begin{aligned} A = A^{(1)} + A^{(2)} + A^{(3)}, \end{aligned}$$

where each $A^{(e)}$ corresponds to a $4 \times 4$ block contribution to the global matrix. Each block arises from a local matrix $\tilde{A}^{(e)} \in \mathbb {R}^{4 \times 4}$. Define restriction matrices $R^{(e)} \in \mathbb {R}^{4 \times 10}$ that extract the relevant global degrees of freedom:

$$\begin{aligned} R^{(1)}= & \begin{bmatrix} 1 & 0 & 0 & 0 & 0 & 0 & 0 & 0 & 0 & 0 \\ 0 & 1 & 0 & 0 & 0 & 0 & 0 & 0 & 0 & 0 \\ 0 & 0 & 1 & 0 & 0 & 0 & 0 & 0 & 0 & 0 \\ 0 & 0 & 0 & 1 & 0 & 0 & 0 & 0 & 0 & 0 \end{bmatrix},\\R^{(2)}= & \begin{bmatrix} 0 & 0 & 0 & 1 & 0 & 0 & 0 & 0 & 0 & 0 \\ 0 & 0 & 0 & 0 & 1 & 0 & 0 & 0 & 0 & 0 \\ 0 & 0 & 0 & 0 & 0 & 1 & 0 & 0 & 0 & 0 \\ 0 & 0 & 0 & 0 & 0 & 0 & 1 & 0 & 0 & 0 \end{bmatrix},\\R^{(3)}= & \begin{bmatrix} 0 & 0 & 0 & 0 & 0 & 0 & 1 & 0 & 0 & 0 \\ 0 & 0 & 0 & 0 & 0 & 0 & 0 & 1 & 0 & 0 \\ 0 & 0 & 0 & 0 & 0 & 0 & 0 & 0 & 1 & 0 \\ 0 & 0 & 0 & 0 & 0 & 0 & 0 & 0 & 0 & 1 \end{bmatrix}. \end{aligned}$$

Each local matrix $\tilde{A}^{(e)} \in \mathbb {R}^{4 \times 4}$ is defined as:

$$\begin{aligned} \tilde{A}^{(e)} = \tilde{M}^{(e)} + \Delta t \cdot \tilde{K}^{(e)}. \end{aligned}$$

The contribution to the global system is then given by:

$$\begin{aligned} A^{(e)} = (R^{(e)})^T \tilde{A}^{(e)} R^{(e)}. \end{aligned}$$

In Algorithm 3 and Algorithm 4, we give additive and multiplicative Schwarz methods respectively for $A = M + \Delta t \cdot K$.

**Algorithm 3:**
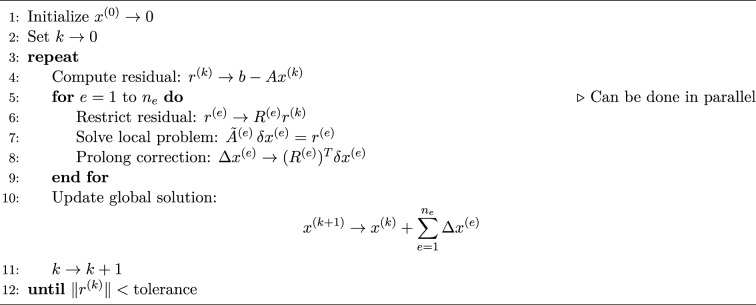
Additive Schwarz Method

**Algorithm 4:**
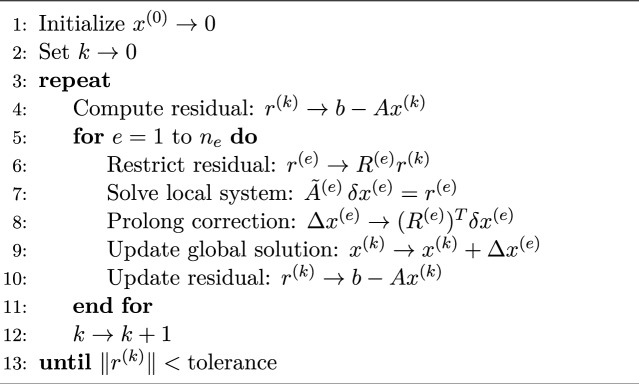
Multiplicative Schwarz Method

The additive Schwarz method enables parallel computation by dividing the global problem into overlapping subdomains, where each subproblem can be solved independently. In each iteration, the solution on each subdomain is computed using the current global estimate, and all subdomain solutions are calculated simultaneously. Once all local problems are solved, their contributions are combined to update the global solution. This independence makes additive Schwarz method highly suitable for distributed computing environments, where multiple processors or cores can work on different parts of the domain at the same time.

In contrast, the multiplicative Schwarz method is inherently sequential. Here, subdomain problems are also solved one at a time, but the key difference is that the solution is updated after each subdomain solve. This means the next subdomain uses the most recent information from the previous update, which often results in faster convergence, similar to the way Gauss-Seidel works compared to Jacobi iteration. However, because each subdomain relies on the result of the previous one, multiplicative Schwarz method cannot be parallelized easily and is typically used in serial computation environments.

## Parallel and sequential quantum algorithm design

Before presenting our proposed parallel and sequential quantum algorithms, we first review the foundational of HHL quantum algorithm^5^. The HHL algorithm provides a quantum approach to solving linear systems with exponential speedup under certain conditions. This algorithm serves as the theoretical foundation for our proposed quantum algorithms.

### Overview of the HHL quantum algorithm

As a result of discretizing the partial differential equation, the problem is reduced to solving a linear system of equations:

$$\begin{aligned} Ax =b, \end{aligned}$$

where $A \in \mathbb {R}^{N \times N}$ is a Hermitian matrix, and $b \in \mathbb {R}^{N}$ is derived from the source term and boundary conditions. Although classical algorithms require polynomial time to solve such systems, quantum computing provides a framework for exponential acceleration through the use of the HHL algorithm^5,21,22,24–26^. The HHL algorithm comprises three main stages: *quantum phase estimation (QPE)*, *controlled rotation*, and *uncomputation*. The process begins by encoding the input vector $b$ into a quantum state $|b\rangle$. QPE is then used to extract the eigenvalues $\lambda _j$ of the matrix $A$, which are stored in an ancillary register. A controlled rotation is subsequently applied to an auxiliary qubit, with rotation angles determined by the reciprocals $1/\lambda _j$. Finally, the inverse QPE (uncomputation) is performed to disentangle the eigenvalue register, resulting in a quantum state $|x\rangle$ that approximates the solution to the linear system.

Let $A$ be a Hermitian matrix with spectral decomposition:

$$\begin{aligned} A = \sum _{j=0}^{N-1} \lambda _j |u_j\rangle \langle u_j|, \end{aligned}$$

where $\lambda _j$ are the eigenvalues and $|u_j\rangle$ are the corresponding orthonormal eigenvectors. Since $A$ is diagonalizable in the eigenbasis $\{ |u_j\rangle \}$, its inverse can be expressed as

$$\begin{aligned} A^{-1} = \sum _{j=0}^{N-1} \lambda _j^{-1} |u_j\rangle \langle u_j|. \end{aligned}$$

Assuming that the input vector $|b\rangle$ admits the decomposition

$$\begin{aligned} |b\rangle = \sum _{j=0}^{N-1} \beta _j |u_j\rangle , \end{aligned}$$

the solution to the linear system is given by

$$\begin{aligned} |x\rangle = A^{-1}|b\rangle = \sum _{j=0}^{N-1} \frac{\beta _j}{\lambda _j} |u_j\rangle , \end{aligned}$$

which the HHL algorithm approximates as a quantum state.

The quantum circuit implementing the HHL algorithm utilizes three main quantum registers. The first register, consisting of $n_b$ qubits, encodes the input vector $b$ as a quantum state $|b\rangle$, and ultimately holds a state proportional to the solution $|x\rangle$. The second register, of size $n_\ell$, stores the binary representation of the eigenvalues obtained via QPE. Additionally, an ancillary qubit is used to perform a controlled rotation, where the rotation angle is inversely related to the estimated eigenvalues. This step effectively simulates the action of the matrix inverse required to solve the linear system. Initially, all qubits are initialized in the $|0\rangle$ state. The state $|b\rangle$ is prepared by applying an appropriate unitary transformation to the $n_b$-qubit register. QPE is then performed using controlled applications of the unitary operator $e^{iAt}$, where $A$ is the Hermitian matrix associated with the system $Ax = b$, and $t$ is a selected evolution time. The resulting eigenvalues $\lambda _j$ are encoded in the $n_\ell$-qubit register. Subsequently, a controlled rotation is applied to the ancillary qubit, conditioned on the contents of the eigenvalue register. This operation maps the amplitude of the ancillary qubit to be proportional to $1/\lambda _j$, thereby encoding the reciprocal of the eigenvalues. Next, inverse quantum phase estimation ($\hbox {QPE}^{-1}$) is applied to uncompute the eigenvalue register and disentangle it from the system. Finally, a measurement is performed on the ancillary qubit. If the measurement outcome is $|1\rangle$, the post-measurement state of the $n_b$-register is proportional to the desired solution $|x\rangle = A^{-1}|b\rangle$. The HHL algorithm consists of the following key subroutines:

1. **State Preparation:** The input vector $b$ is normalized and encoded into a quantum state $\vert {b} \rangle$ expressed in the eigenbasis $\{ \vert {u_j} \rangle \}$ of matrix $A$:
  $$\begin{aligned} {\left| 0 \right\rangle _{{n_b}}} \rightarrow {\left| b \right\rangle _{{n_b}}} = \sum \limits _j {{\beta _j}} \left| {{u_j}} \right\rangle ,\quad \textrm{where}\,\,{\beta _j} = \langle {u_j}|b\rangle . \end{aligned}$$
2. **Quantum Phase Estimation (QPE):** This step estimates the eigenvalues $\lambda _j$ associated with eigenvectors $\vert {u_j} \rangle$ by applying controlled time evolution $e^{iAt}$, followed by an inverse quantum Fourier transform (QFT$^{-1}$) on the ancilla register. The resulting state is:
  $$\begin{aligned} \sum _j \beta _j \vert {\lambda _j} \rangle _{n_\ell } \otimes \vert {u_j} \rangle _{n_b}, \end{aligned}$$
  where $n_\ell$ denotes the number of qubits used to represent eigenvalues with finite precision.
3. **Eigenvalue Inversion via Controlled Rotation:** A controlled rotation is applied to an ancillary qubit based on the estimated eigenvalues, encoding the reciprocal $1/\lambda _j$ into the amplitude:
  $$\begin{aligned} \sum _j \beta _j \vert {\lambda _j} \rangle \otimes \vert {u_j} \rangle \otimes \left( \sqrt{1 - \frac{C^2}{\lambda _j^2}} \vert {0} \rangle + \frac{C}{\lambda _j} \vert {1} \rangle \right) , \end{aligned}$$
  where $C$ is a scaling constant such that $C \le \min _j |\lambda _j|$, ensuring valid rotation angles.
4. **Uncomputation:** The QPE operation is reversed to disentangle the eigenvalue register:
  $$\begin{aligned} \sum _j \beta _j \vert {0} \rangle _{n_\ell } \otimes \vert {u_j} \rangle \otimes \left( \sqrt{1 - \frac{C^2}{\lambda _j^2}} \vert {0} \rangle + \frac{C}{\lambda _j} \vert {1} \rangle \right) . \end{aligned}$$
5. **Measurement:** Measuring the final ancilla qubit and obtaining outcome $\vert {1} \rangle$ collapses the state to one proportional to the solution vector $\vert {x} \rangle = A^{-1} \vert {b} \rangle$:
  $$\begin{aligned} \vert {\tilde{x}} \rangle \propto \sum _j \frac{\beta _j}{\lambda _j} \vert {u_j} \rangle , \end{aligned}$$
  which approximates the desired result up to normalization. The process may be repeated until this outcome is observed.
6. **Post-Processing:** If an observable $M$ is of interest, its expectation value with respect to $\vert {x} \rangle$ can be computed as:
  $$\begin{aligned} \langle x | M | x \rangle . \end{aligned}$$

### Parallel quantum algorithm

**Fig. 2:**
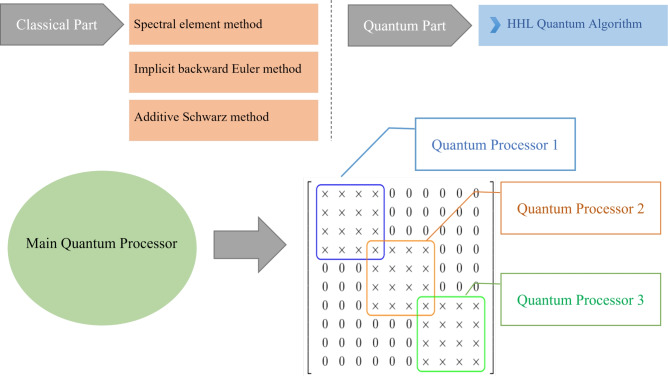
Schematic of parallel quantum algorithm.

In this subsection, we present a parallel quantum algorithm for solving the partial differential equation (1)–(3). Also in Fig. 2 the schematic of the parallel quantum algorithm is given.
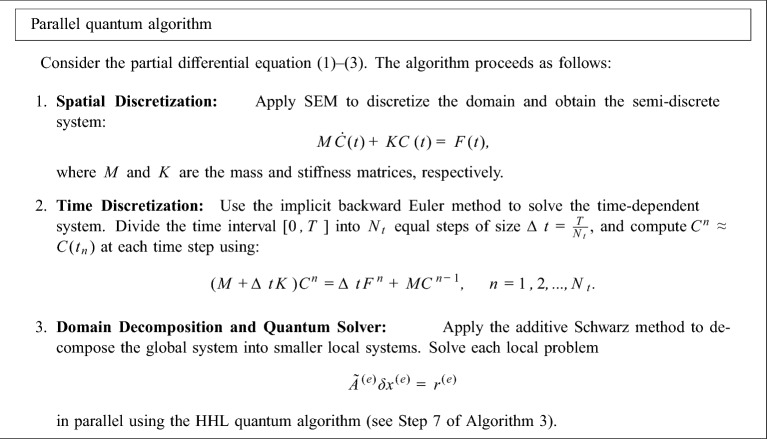


### Sequential quantum algorithm

In this subsection, we present a sequential quantum algorithm for solving the partial differential equation (1)–(3). Also in Fig. 2 the schematic of the sequential quantum algorithm is given.
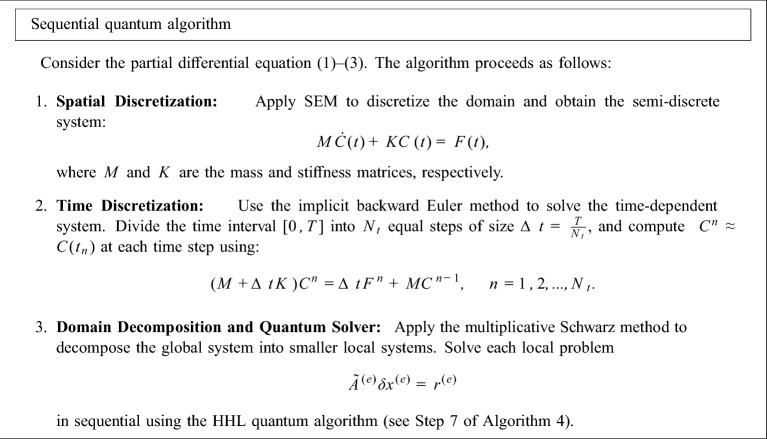


**Fig. 3:**
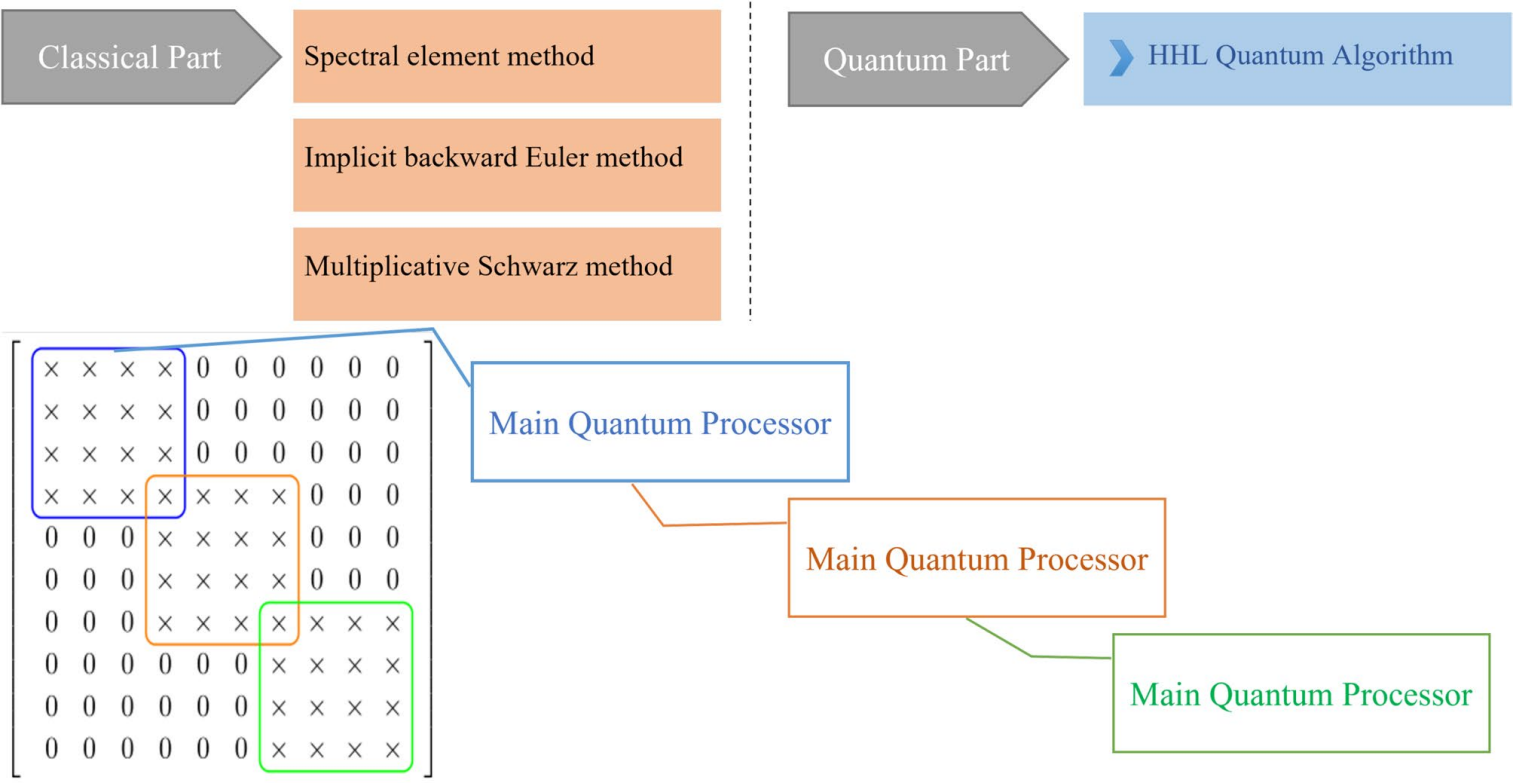
Schematic of sequential quantum algorithm.

### HHL output and result composition

The HHL algorithm outputs a quantum state

$$|x\rangle = \frac{A^{-1}|b\rangle }{\Vert A^{-1}|b\rangle \Vert },$$

which encodes the normalized classical solution vector of the linear system $A x = b$ in its probability amplitudes. During computation, the ancilla qubit in the HHL circuit controls the eigenvalue inversion process. Measuring the ancilla in the $|1\rangle$ state collapses the system qubits into a state proportional to the desired solution:

$$|\tilde{x}\rangle \propto \sum _j \frac{\beta _j}{\lambda _j} |u_j\rangle ,$$

where $\lambda _j$ and $|u_j\rangle$ denote the eigenvalues and eigenvectors of $A$, and $\beta _j = \langle u_j | b \rangle$. Since this measurement is probabilistic, multiple repetitions may be required to obtain the $|1\rangle$ outcome. The resulting quantum state encodes the solution information in its amplitudes, which cannot be directly accessed by a single measurement. Instead, properties of $|\tilde{x}\rangle$ are inferred through *quantum state tomography* or by estimating expectation values of specific observables, depending on the hardware capabilities and target quantities.

#### Quantum state tomography

Full quantum state tomography enables comprehensive characterization of the quantum state produced by the HHL algorithm through a series of projective measurements performed in multiple bases. By repeatedly measuring identically prepared instances of the output state $|x\rangle$ in complementary bases associated with the Pauli operators $X$, $Y$ and $Z$, the corresponding density matrix can be reconstructed as:

$$\rho = |x\rangle \langle x|.$$

The reconstructed density matrix reveals both population probabilities and coherence terms. From these quantities, the amplitude coefficients $\{x_i\}$ of the classical solution vector can be extracted. For small-scale systems, full tomography is practical and allows accurate verification of the quantum solution. For larger systems, specific expectation values can be efficiently estimated using techniques such as quantum amplitude estimation, without requiring full state reconstruction.

On NISQ devices, tomography is used mainly to characterize small systems of up to about 5–10 qubits. In fault-tolerant architectures, observables such as $\langle x | M | x \rangle$ can be obtained more efficiently using amplitude estimation or other protocols, eliminating the need for full tomography. Also, in some classical simulation environments (e.g., Mathematica’s QuantumCircuitOperator), the full amplitude vector can be directly extracted and normalized to recover the classical solution $x = A^{-1}b$.

#### Result composition and post-processing

After obtaining the reconstructed local solutions $\delta x^{(e)}$ from each HHL quantum solver, the results are assembled through a classical post-processing step following the domain decomposition strategy. In the parallel quantum algorithm, all subdomain corrections are combined simultaneously using the additive Schwarz update rule:

$$x^{(k+1)} = x^{(k)} + \sum _{e=1}^{n_e} \Delta x^{(e)}.$$

This formulation preserves global continuity while enabling concurrent quantum computation across distributed subdomains, providing a scalable approach for parallel implementation on multiple quantum processors.

In contrast, the sequential quantum algorithm employs the multiplicative Schwarz strategy, where each subdomain solution is incorporated into the global approximation immediately after computation. The update is performed as:

$$x^{(k)} \rightarrow x^{(k)} + \Delta x^{(e)}, \qquad r^{(k)} \rightarrow b - A x^{(k)}.$$

This sequential process ensures that boundary corrections are progressively propagated through the domain, leading to improved numerical stability and faster convergence in strongly coupled systems.

#### Density matrix reconstruction for the two-qubit HHL output

**Fig. 4:**
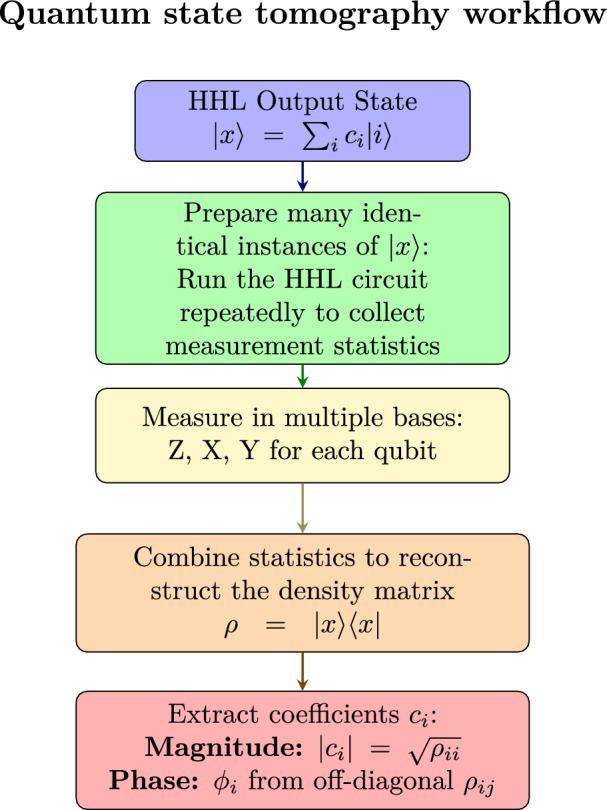
Schematic of full quantum state tomography for reconstructing the coefficients $c_i$ of a 2-qubit HHL output state.

For the two-qubit HHL implementation, full quantum state tomography reconstructs the complete density matrix $\rho$ in the Pauli basis:

$$\rho = \frac{1}{4} \sum _{i,j=0}^{3} \langle \sigma _i \otimes \sigma _j \rangle \, (\sigma _i \otimes \sigma _j),$$

where $\sigma _0 = I, \sigma _1 = X, \sigma _2 = Y, \sigma _3 = Z$ and $\langle \sigma _i \otimes \sigma _j \rangle = \textrm{Tr}[\rho (\sigma _i \otimes \sigma _j)]$ are the measured expectation values. Projective measurements in multiple bases on identically prepared copies of $|x\rangle$ allow reconstruction of both diagonal and off-diagonal elements, capturing population probabilities and coherence terms. Using these statistics, the reconstructed density matrix fully characterizes the quantum state, enabling comparison with the theoretical prediction $|x\rangle \langle x|$ and validation of the HHL implementation’s accuracy and fidelity (see Fig. 4).

In next section, the application of the proposed algorithms to solve the problem (1)–(3) is shown by an example.

## Numerical results

To demonstrate the efficiency of the proposed algorithms, we present a numerical example in this section. Let the final time be $T = 1$, and set the number of time steps to $N_t = 10$, yielding a time step size of $\Delta t = 0.1$. For simplicity, we compute only the first time step $C^1 \approx C(t_1) = C(0.1)$. Specifically, we solve the following linear system:

$$(M + 0.1\, K) C^1 = 0.1\, F^1 + M C^0,$$

using both the proposed parallel and sequential quantum algorithms. In SEM, the partial differential equation (1)–(3) is discretized over $n_e = 3$ elements, and the degree of the Legendre polynomial basis is chosen as $N = 3$. Also, classical computations were performed using *Mathematica 14.0* on a desktop computer equipped with a Pentium(R) Dual-Core CPU E5700 @ 3.00 GHz. The quantum implementation of the proposed algorithm was conducted using the IBM Quantum simulator, accessed through the Qiskit framework. Qiskit is an open-source quantum computing software development kit that provides tools for designing, simulating, and executing quantum algorithms on both simulators and real quantum hardware. The use of the IBM simulator offers a reliable and noise-free environment for evaluating the behavior of quantum circuits, ensuring that the algorithm operates as theoretically expected. This simulation environment plays a crucial role in the development pipeline, as it allows for extensive testing and debugging of the quantum algorithm prior to execution on actual quantum devices, which are currently limited by noise, decoherence, and qubit connectivity constraints.

Now, consider the partial differential equation (1)–(3), where the exact solution is:

$$\begin{aligned} u_{\text {exact}}(x,t) = \left( {t + 1} \right) \sin \left( {\pi x} \right) \sin \left( {\pi \left( {1 - x} \right) } \right) . \end{aligned}$$

So, we have $\phi (x)= u_{\text {exact}}(x,0)=\sin \left( {\pi x} \right) \sin \left( {\pi \left( {1 - x} \right) } \right)$. Applying SEM, the matirx $M + 0.1\, K$ is obtained by:

$$\begin{aligned} \left( \begin{array}{cccccccccc} 1.327 & -1.463 & 0.213 & -0.050 & 0 & 0 & 0 & 0 & 0 & 0 \\ -1.463 & 2.638 & -1.250 & 0.213 & 0 & 0 & 0 & 0 & 0 & 0 \\ 0.213 & -1.25 & 2.638 & -1.463 & 0 & 0 & 0 & 0 & 0 & 0 \\ -0.050 & 0.213 & -1.463 & 2.655 & -1.463 & 0.213 & -0.050 & 0 & 0 & 0 \\ 0 & 0 & 0 & -1.463 & 2.638 & -1.25 & 0.213 & 0 & 0 & 0 \\ 0 & 0 & 0 & 0.213 & -1.25 & 2.638 & -1.463 & 0 & 0 & 0 \\ 0& 0 & 0 & -0.05 & 0.213 & -1.463 & 2.655 & -1.463 & 0.213 & -0.050 \\ 0 & 0 & 0 & 0 & 0 & 0 & -1.463 & 2.638 & -1.25 & 0.213 \\ 0 & 0 & 0 & 0. & 0 & 0 & 0.213 & -1.25 & 2.638 & -1.463 \\ 0& 0& 0 & 0 & 0 & 0 & -0.05 & 0.213 & -1.463 & 1.327 \\ \end{array} \right) \end{aligned}$$

and ${\left( {0.1{\hspace{0.55542pt}} {F^1} + M{C^0}} \right) ^T}$ is obtained by

$$\begin{aligned} \left( \begin{array}{cccccccccc} -0.0621&-0.235&0.050&0.107&0.414&0.414&0.107&0.050&-0.235&-0.062 \end{array} \right) . \end{aligned}$$

Now, we apply the proposed parallel and sequential quantum algorithms. The $L_{2}$-norm errors and the relative errors of the proposed method for 10 iterations in the additive and multiplicative Schwarz methods are listed in Table 1. For the function *u*(*x*, *t*) defined on the domain $\Omega = (0,1)$, the $L_2$-norm error at time $t = 0.1$ is defined as:

$$E_{L_2} = \Vert u_{\text {exact}}(\cdot , 0.1) - u_{\text {HHL}}(\cdot , 0.1) \Vert _{L_2(\Omega )} = \left( \int _{\Omega } \left| u_{\text {exact}}(x,0.1) - u_{\text {HHL}}(x,0.1) \right| ^2 dx \right) ^{1/2},$$

and the corresponding relative $L_2$ error is

$$E_{\text {rel}} = \frac{\Vert u_{\text {exact}}(\cdot ,0.1) - u_{\text {HHL}}(\cdot ,0.1) \Vert _{L_2(\Omega )}}{\Vert u_{\text {exact}}(\cdot ,0.1) \Vert _{L_2(\Omega )}}.$$

**Fig. 5:**
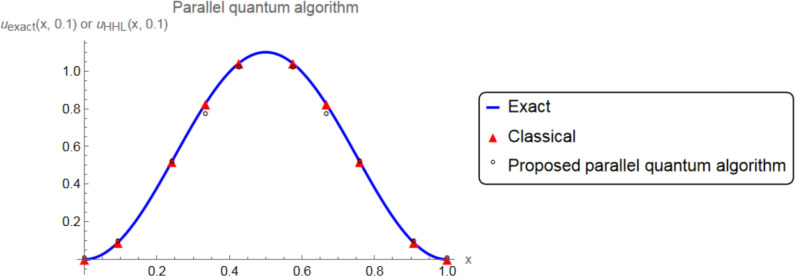
The plots of exact and the approximate solutions with the proposed parallel quantum algorithm.

**Fig. 6:**
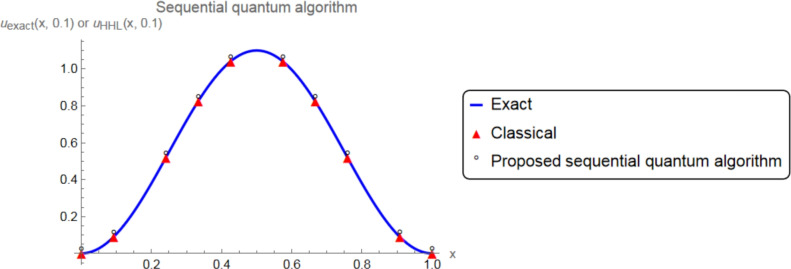
The plots of exact and the approximate solutions with the proposed sequential quantum algorithm.

Here, $u_{\text {exact}}$ is the exact solution of the PDE and $u_{\text {HHL}}$ is the solution obtained from the HHL-based quantum algorithm. In addition, the exact solution $u_{\text {exact}}(x,t) = \left( {t + 1} \right) \sin \left( {\pi x} \right) \sin \left( {\pi \left( {1 - x} \right) } \right)$ and the approximate solution for $t=0.1$ is demonstrated in Figs. 5 and 6 with the proposed parallel and sequential quantum algorithms, respectively. In these figures and table, we clearly observe that the desired solution is provided by the approximate solution as well.

**Table 1:** The $L_2$-norm errors and the relative errors of the proposed method.

	$N_{t}$	*N*	Method	Error
$L_2$-norm error	10	3	Classical additive Schwarz method	$1.41\times 10^{-1}$
	10	3	The parallel quantum algorithm	$1.36\times 10^{-1}$
	10	3	Classical multiplicative Schwarz method	$5.77\times 10^{-3}$
	10	3	The sequential quantum algorithm	$2.13\times 10^{-3}$
Relative error	10	3	Classical additive Schwarz method	$6.99\times 10^{-2}$
	10	3	The parallel quantum algorithm	$6.83\times 10^{-2}$
	10	3	Classical multiplicative Schwarz method	$2.85\times 10^{-3}$
	10	3	The sequential quantum algorithm	$7.22\times 10^{-4}$

**Fig. 7:**
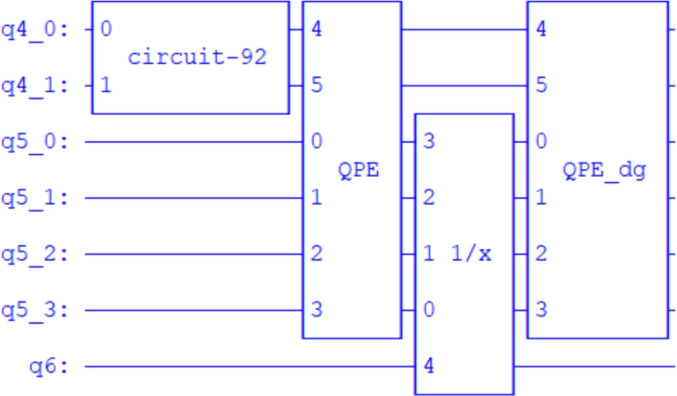
The circuit implements the HHL quantum algorithm. Circuit-92 performs the state preparation using amplitude encoding, initializing the quantum state $|b \rangle$. The qubits $q4\_0$ and $q4\_1$ form the state register that stores $|b \rangle$, while *q*6 serves as an ancilla qubit for controlled rotations. The qubits $q5\_0, q5\_1, q5\_2,$ and $q5\_3$ make up the eigenvalue register, which holds the results of the Quantum Phase Estimation (QPE) subroutine. The 1/*x* gate implements eigenvalue inversion and $\mathrm{QPE\_dg}$ denotes the inverse (dagger) of QPE, which uncomputes the eigenvalue register to disentangle it from the state register.

The quantum circuit implementation of the HHL algorithm required a total of 7 qubits (see Fig. 7), allocated across three primary roles: eigenvalue encoding via quantum phase estimation, state representation of the input vector, and an ancillary qubit for the controlled rotation step. In the context of the proposed parallel and sequential quantum algorithms, this qubit structure serves as a foundational unit. For the parallel quantum algorithm, multiple HHL circuits can be executed simultaneously across distributed quantum nodes, each solving a local subdomain system as dictated by the additive Schwarz method. Each node replicates the 7-qubit configuration independently, enabling concurrent processing and efficient scalability. In contrast, the sequential quantum algorithm, aligned with the multiplicative Schwarz method, reuses a single HHL circuit structure iteratively. The state is updated after each subdomain solve and passed to the next, preserving inter-domain dependencies. This reuse minimizes qubit overhead while maintaining consistency across the computational domains. Together, these strategies highlight how the modular HHL circuit can be adapted to support scalable, domain-decomposed quantum solutions to large linear systems.

## Conclusion

This paper studied one of the most pressing challenges in applying quantum algorithms to scientific computing: the limited number of qubits available in near-term quantum processors. Instead of requiring a large quantum system to solve global problems directly, we introduced a distributed strategy that decomposes the global system into smaller subdomain problems, each solvable with far fewer qubits. By integrating the spectral element method with both additive and multiplicative Schwarz domain decomposition methods, we developed quantum-compatible formulations that map effectively to parallel and sequential execution models, respectively.

Notably, each subproblem fits within the modest qubit capacities of current quantum hardware. This architecture allows multiple HHL-based solvers to run in parallel across quantum nodes (in the additive case) or iteratively on a single device (in the multiplicative case), making full use of limited quantum resources without sacrificing the accuracy and convergence properties of high-order spectral methods.

Rather than being a theoretical abstraction, the proposed method offers a realistic and scalable pathway toward applying quantum algorithms to large-scale partial differential equations. As quantum hardware matures, the modular and qubit-efficient nature of this framework makes it well-positioned for early adoption in hybrid high-performance computing environments.

## Data Availability

The datasets used and/or analysed during the current study available from the corresponding author on reasonable request.

## References

[1] Patera, A. T. A spectral element method for fluid dynamics: Laminar flow in a channel expansion. *Journal of Computational Physics* **54**(3), 468–488 (1984).

[2] Karniadakis, G. E. & Sherwin, S. J. *Spectral/hp Element Methods for Computational Fluid Dynamics* 2nd edn. (Oxford University Press, 2005).

[3] Moazzezi, S., Salehi Shayegan, A. H. & Zakeri, A. A spectral element method for solving backward parabolic problems. *International Journal for Computational Methods in Engineering Science and Mechanics* **21**(1), 45–54 (2020).

[4] Salehi Shayegan, A. H. & Zakeri, A. Quasi solution of a backward space fractional diffusion equation. *Journal of Inverse and Ill-posed Problems* **27**(6), 795–814 (2019).

[5] Harrow, A. W., Hassidim, A. & Lloyd, S. Quantum algorithm for linear systems of equations. *Physical Review Letters* **103**(15), 150502 (2009). 19905613 10.1103/PhysRevLett.103.150502

[6] Cuomo, D., Caleffi, M. & Cacciapuoti, A. S. Towards a distributed quantum computing ecosystem. *IET Quantum Communication* **1**(1), 3–8 (2020).

[7] Gyongyosi, L. & Imre, S. A survey on quantum computing technology. *Computer Science Review* **31**, 51–71 (2019).

[8] Smith, B. F., Bjorstad, P. E. & Gropp, W. D. *Domain Decomposition: Parallel Multilevel Methods for Elliptic Partial Differential Equations* (Cambridge University Press, 1996).

[9] Canuto, C., Hussaini, M. Y., Quarteroni, A. & Zang, T. A. *Spectral Methods in Fluid Dynamics* (Springer, 1986).

[10] van de Vosse, F. N. & Minev, P. *Spectral Element Methods: Theory and Applications*,Eindhoven University of Technology Report, ISBN 90-236, (1996).

[11] Zienkiewicz, O. C. & Taylor, R. L. *The Finite Element Method* 5th edn, Vol. 1:The Basis, (Butterworth-Heinemann, Oxford, 2000).

[12] Quarteroni, A., Sacco, R. & Saleri, F. *Numerical Mathematics* 2nd edn. (Springer, 2007).

[13] Kimble, H. J. The quantum internet. *Nature* **453**(7198), 1023–1030 (2008). 18563153 10.1038/nature07127

[14] Cirac, J. I., Zoller, P., Kimble, H. J. & Mabuchi, H. Distributed quantum computation over noisy channels. *Physical Review Letters* **78**(16), 3221 (1997).

[15] Van Meter, R. *Architecture of a quantum multicomputer optimized for Shor’s factoring algorithm* (Springer, 2016).

[16] Wehner, S., Elkouss, D. & Hanson, R. Quantum internet: A vision for the road ahead. *Science* **362**(6412), eaam9288 (2018). 30337383 10.1126/science.aam9288

[17] Monroe, C. & Kim, J. Large-scale modular quantum-computer architecture with atomic memory and photonic interconnects. *Science* **339**(6124), 1164–1169 (2013). 23471398 10.1126/science.1231298

[18] Acín, A. et al. Quantum technologies: The second quantum revolution. *New Journal of Physics* **20**(8), 080201 (2018).

[19] Elliott, C. Quantum key distribution and cryptography. *IEEE Communications Magazine* **34**(4), 28–34 (1996).

[20] Schwarz, H. A. Über einige Abbildungsaufgaben. *Journal für die reine und angewandte Mathematik* **70**, 105–120 (1870).

[21] Childs, A. M., Kothari, R. & Somma, R. D. Quantum linear systems algorithm with exponentially improved dependence on precision. *SIAM J. Comput.* **46**(6), 1920–1950 (2017).

[22] Cao, Y., Papageorgiou, A. & Kais, S. Quantum algorithm and circuit design solving the Poisson equation. *New Journal of Physics* **15**(1), 013021 (2013).

[23] Clader, B. D., Jacobs, B. C. & Sprouse, C. R. Preconditioned quantum linear system algorithm. *Physical Review Letters* **110**(25), 250504 (2013). 23829722 10.1103/PhysRevLett.110.250504

[24] Childs, A. M., Kothari, R. & Somma, R. D. Quantum algorithm for systems of linear equations with exponentially improved dependence on precision. *SIAM Journal on Computing* **46**(6), 1920–1950 (2017).

[25] Biamonte, J. et al. Quantum machine learning. *Nature* **549**(7671), 195–202 (2017). 28905917 10.1038/nature23474

[26] Salehi Shayegan, A. H. & Dejam, L. Quantum linear system algorithm for solving an ill-posed quasi-linear elliptic problem by preconditioning operator. *European Physical Journal Plus* **140**(5), 1–14 (2025).

